# Quantifying culture gaps between physicians and managers in Dutch hospitals: a survey

**DOI:** 10.1186/1472-6963-10-86

**Published:** 2010-04-01

**Authors:** Hanneke AHJ Klopper-Kes, Sabine Siesling, Nienke Meerdink, Celeste PM Wilderom, Wim H van Harten

**Affiliations:** 1 Ziekenhuisgroep Twente, Almelo, The Netherlands; 2 School of Management and Governance, University of Twente, Enschede, the Netherlands; 3 Comprehensive Cancer Centre North East, Enschede/Groningen, the Netherlands; 4 Netherlands Cancer Institute/Antoni van Leeuwenhoek Hospital, Amsterdam, the Netherlands

## Abstract

**Background:**

The demands in hospitals for safety and quality, combined with limitations in financing health care require effective cooperation between physicians and managers. The complex relationship between both groups has been described in literature. We aim to add a perspective to literature, by developing a questionnaire which provides an opportunity to quantitatively report and elaborate on the size and content of differences between physicians and managers. Insight gained from use of the questionnaire might enable us to reflect on these differences and could provide practical tools to improve cooperation between physicians and managers, with an aim to enhance hospital performance.

**Methods:**

The CG-Questionnaire was developed by adjusting, pre-testing, and shortening Kralewski's questionnaire, and appeared suitable to measure culture gaps. It was shortened by exploratory factor analysis, using principal-axis factoring extraction with Varimax rotation. The CG-Questionnaire was sent to all physicians and managers within 37 Dutch general hospitals. ANOVA and paired sample T-tests were used to determine significant differences between perceptions of daily work practices based in both professional cultures; culture gaps. The size and content of culture gaps were determined with descriptive statistics.

**Results:**

The total response (27%) consisted of 929 physicians and 310 managers. The Cronbachs alpha's were 0.70 - 0.79. Statistical analyses showed many differences; culture gaps were found in the present situation; they were even larger in the preferred situation. Differences between both groups can be classified into three categories: (1) culture gaps in the present situation and not in the preferred, (2) culture gaps in the preferred situation and not in the present, and (3) culture gaps in both situations.

**Conclusions:**

With data from the CG-Questionnaire it is now possible to measure the size and content of culture gaps between physicians and managers in hospitals. Results gained with the CG-Questionnaire enables hospitals to reflect on these differences. Combining the results, we distinguished three categories of increasing complexity. We linked these three categories to three methods from intergroup literature (enhanced information, contact and ultimately meta cognition) which could help to improve the cooperation between physicians and managers.

## Background

The history of the development of hospital organizations and the wider scope of quality initiatives shows that cooperation between physicians and managers is becoming of paramount importance to enhance hospital performance [1]. Hospitals are charged with developing internal organizations where solid quality and cost effectiveness go hand in hand [2-4]. More and more quality initiatives are being promoted in the public domain (100,000 and 5 Million Lives Campaigns IHI, 2006-2008, and the report on quality "Crossing the Quality Chasm" published by the US Institute of Medicine [5]) which increases the demand of patients for higher transparency in the quality of care. Moreover, new treatments are made possible by technological innovations, resulting in improved opportunities to cure diseases. However, budgets are under strain and the limitations of collective health care financing become apparent. Physicians increasingly have to work closely together with managers and have to negotiate for resources and the organization of their clinical practices [1]. New quality (management) techniques (integrating financial and quality management) have been initiated to meet the aforementioned challenges in the hospital organization [6]. These quality initiatives usually do not take professional standards into account and often lead to an increasing influence of managers over quality and efficiency measures that influences the work done by physicians. One of the consequences is that physicians may feel that their autonomy is being threatened and resist the implementation [7].

In the hospital setting there are well known difficulties in the cooperation between physicians and managers [7-11]. The cooperation is even more complicated because of the differences in professional cultures between both groups [8]. Given the described complex context, insight is needed into the content and size of the differences between physicians and managers. Theoretically, the concept of professional culture differences between physicians and managers is apparent in various ways. Schein [12] conceptualizes organizational culture into three layers: basic assumptions, artifacts, and values and beliefs. The basic assumptions are mainly implicit and therefore not directly measurable by quantitative means. The artifacts are the distinguishable expressions of organizational culture. When you walk into a hospital, the different professional groups are immediately apparent. For example, physicians almost always wear their white coats and stethoscopes, some even during lunch or management meetings. This distinguishes them from all other groups in the hospital. Managers are less visible with regard to their appearances. They do, however, differ from physicians, for example, in their use of language (management jargon) which is very often not understood by physicians [13]. The operationalization of artifacts is mainly applicable for qualitative research purposes. We wanted to study the differences between physicians and managers quantitatively; when collecting our data we asked for perceptions of daily practices which were based on Schein's third culture layer: values and beliefs. According to Hofstede et al. [14] the largest part of a firm's culture is 'organizational practices'. Organizational practices reflect the collective wisdom within an organization as to how things can best be done. Practices are a key visible part of culture. We refer to different perceptions of practices, based on the inherent professional culture dissimilarities between physicians and managers [8], as culture gaps. The complex cooperation based on differences in professional cultures between physicians and managers combined with the fact that both groups are working within the same organizational setting, can be seen as an intergroup conflict setting [15]. From intergroup literature we know that larger differences between groups correlate with reduced performances [16-20]. In intergroup conflict settings, people tend to exaggerate differences between both groups, leading to stereotyping [21]. When a group has stereotypical beliefs about another group, information is filtered towards the stereotypical image of the other group, leading to negative images. In this process, it is very hard to work with an open attitude with the other group. Intergroup literature also provides methods to enhance cooperation in intergroup conflict situations [22-25].

### Measurement of culture gaps

We based our CG-Questionnaire on Kralewski's work [26,27] on the culture of medical group practices. It uses underlying issues in health care (such as the need for more efficacy, safety, and quality of care) that are major issues in all western countries [28]. Kralewski et al. [27] identified nine relevant organizational culture dimensions in medical group practices. Kralewski's questionnaire has been used among medical group practices, but originated from Reynolds [29] which reviewed five major publications about organizational cultures in industry. After several field studies, Kralewski et al. refined their initial culture instrument into a questionnaire on culture in medical group practices [26]. The questionnaire was validated by a large sample of 267 medical group practices, and contains the following nine dimensions: "collegiality", "information emphasis", "quality emphasis", "organizational identity", "cohesiveness", "business emphasis", "organizational trust", "innovation", and "autonomy". Kaissi et al. [30] used Kralewski's questionnaire to study the influence of culture of medical group practices on types of quality programs used. Smalarz [31] used the survey instrument to study the effect of physician group cultural dimensions on quality performance indicators. In all the studies, Kralewski's questionnaire appeared relevant, reliable, and valid to measure organizational culture within and between hospitals. It captures the culture of medical group practices at an organizational level and was used as the starting point for our study.

An extra feature was added to the Kralewski questionnaire by asking about the present and preferred situation. When asking about the way practices are perceived in the present situation, we searched for the negotiated order between physicians and managers. Answers to statements in the present situation represent organizational reality as perceived by physicians and managers. We postulated that differences in answers in the present situation between the two professional groups reveal culture gaps in the way both groups perceive reality. Additionally, answers to statements in the preferred situation represent the desired order [32]. We postulate that differences in answers in the preferred situation between physicians and managers reveal culture gaps, based on their inherent professional cultures. If culture gaps in the preferred situation are larger compared to the present situation, it could mean that physicians and managers would change the system when acting without the other party. In this case there is a latent conflict between physicians and managers. The larger the size of the culture gaps the larger the latent conflict between both professional groups.

This study adds a perspective to the literature, from a methodological point of view, to improve insight into the complex relationship between physicians and managers. We applied the questionnaire by Kralewski et al. (further referred to as "Kralewski's questionnaire") [26] to the hospital setting and developed the CG-Questionnaire which is suitable to measure the size and content of differences between physicians and managers as culture gaps. In intergroup literature, the explanatory effect of differences between (professional) groups has been shown in many settings [17,20,23-25], and might be of use for the hospital setting as well. Insight gained from the data from the questionnaire might enable us to reflect on the differences and could provide practical tools with which the cooperation between physicians and managers could be improved, ultimately as a benefit for a hospital's performance.

## Methods

In this paper we report the development of the CG-Questionnaire as well as the results from our use of this instrument to assess culture gaps between physicians and managers in a representative sample of Dutch general hospitals. To obtain the national data, we split the research into three separate studies. Firstly we developed a Dutch questionnaire by translating and slightly adapting Kralewski's [26] questionnaire on the cultures of medical group practices and we pre-tested it. Secondly, we sent the resulting adjusted Dutch questionnaire to 445 physicians and 137 managers in five different Dutch general hospitals. With the collected data we carried out statistical analyses to then develop the CG-Questionnaire. Thirdly, in order to obtain nationally representative data, we sent the CG-Questionnaire to all physicians (n = 3941) and managers (n = 680) in 37 Dutch general hospitals. This study does not require ethical approval. The three successive studies are presented below.

### Qualitative pretest, study 1

We translated and back translated Kralewski's culture questionnaire; then Kralewski's statements had to be aligned with the Dutch system features. No changes were needed in the original statements related to the hospital setting. The only two alterations were related to the differences between American and Dutch health care systems. Most hospitals in the Netherlands are non-profit foundations. It is only recently that market elements have been introduced, such as free negotiations on the price of 20% of the volume of hospital care and facilitating new market entries. The majority of physicians is not employed by the hospital, but is associated with a hospital (usually one) and the physicians are partners in their own within-hospital firm. In the within-hospital firms the accumulated fees are divided. Physicians depend on hospital policies for the allocation of staff (for example secretaries and nurses) and equipment. In the Dutch structure physicians have economic autonomy because of the way they are paid, but they are economically dependent on operational and staff support in the hospital. Physicians are organized as the "medical staff", commonly without hierarchy, and they mandate an internal board which has to be consulted on important organizational and medical issues by the executive hospital board. Following from the above we altered two survey statements in the dimension "business emphasis" linked to the financial system, focusing to a lesser degree on commercialization in hospital care. In the Dutch system the degree of market regulation is expected to remain considerably higher than the situation in the United States. The statements which we changed were:

• "Maximizing revenue is our highest priority" into: "Reducing costs is our highest priority".

• "We won't add a piece of equipment if it won't make money" into: "When purchasing medical equipment financial considerations are an important factor".

In order to have six statements per dimension, we added 15 statements concerning work practices which conform to medical protocols, multidisciplinary cooperation, working atmosphere, and the involvement of physicians in the policy and the mission of the organization. Because our goal is different from Kralewski et al., we added a dimension, also consisting of six statements, directly assessing the level of cooperation between physicians and managers and, accordingly, the extent to which there are culture gaps.

For our qualitative pretest we selected 15 representative respondents from four Dutch general hospitals, located in both rural and urban settings, and asked physicians in both surgical and internal medical specialties as well as board members and managers in different hierarchical positions. We piloted the questionnaire by asking the respondents to complete it, in our presence. The Dutch pretesting questionnaire contained 60 statements with a double five-point Likert scale, which assessed the respondents' personal perceptions of practices in the hospital in the present and preferred situation. During the interviews we posed a set of 13 questions about the clarity, redundancy, lay out, relevance, and other format-related aspects of the Dutch questionnaire. If a respondent criticized one of the statements, it meant that the questionnaire was not completely clear. After every interview we improved the clarity of the language used in the Dutch questionnaire, until there were no further such remarks.

### Development of the Culture Gaps Questionnaire, study 2

#### Methodology

An important criterion for a usable questionnaire is that it needs to be short in order to receive better response rates [33]. We sent the adjusted questionnaire to five different general hospitals in the Netherlands (445 physicians and 137 managers). These hospitals were chosen for their geographical location and size in order to cover differences between urban and rural areas and small, medium, and large general hospitals. We analyzed the data of the respondents, 166 physicians (response rate 37%), and 71 managers (response rate 52%), with future usage and shortening in mind. We checked for potential selectivity and found that the division between physicians and managers regarding the demographic characteristics is comparable to the original sample. Therefore we feel the group of respondents reflects the total group. We shortened the questionnaire mainly through an exploratory factor analysis, using principal-axis factoring extraction with Varimax rotation. The adequate (smallest) number of factors was determined by using the Eigenvalue criteria. Factors having an Eigenvalue larger than 1.0 were included. A loading above 0.50 was chosen as the criterion of acceptance as a factor for the present perceptions and above 0.60 for the preferred perceptions. In the case of cross loadings (in two different factors) the highest loading after rotation was taken. The reliability of the adjusted Dutch questionnaire was determined using the Cronbach's alpha. We considered a score above 0.65 to implicate a sufficient internal consistency.

In order to shorten the questionnaire we determined the reliability and the correlation between the list with and without a certain statement with an item-total correlation test. The item-total correlation was the correlation between the item and the sum score calculated without that particular item. If the item-total correlation was below 0.20, the statement was removed from the list. The item with the lowest score was deleted first, and then the item-total correlation was calculated again. This was repeated until all item-total correlations were above 0.20. Subsequently we determined the Cronbach's alpha score. Repeating the factor analysis with the shortened list, the total variance explained was determined. In order to finalize a valid, reliable and relevant version of the CG-Questionnaire, we compared the outcomes of the analyses of all four categories (physicians/managers and present/preferred situation, assuming the factor structure in all categories was the same) with the statements found in the item-total correlation test. When a statement scored in at least three categories, it was included in the CG-Questionnaire. To statistically finalize the shortening of our list we combined the statements that had been identified with the factor analysis (with Varimax rotation) with the statements which revealed significant differences (ANOVA). We took at least two statements per initial Kralewski dimension that scored significant culture gaps in at least one of the five hospitals. If only one statement scored in a dimension, based on the factor analysis, we took a second statement based on the culture gaps scores.

#### Results

Table 1 presents the results of the factor analyses and the item-total correlations. The Cronbach's alphas for the four different groups varied from 0.87 to 0.90. The factor analysis revealed 19 to 20 components, with different components per group. For the present situation we selected statements with loadings above 0.50 for the present situation which resulted in 42 statements for the physicians and 43 statements for the managers. In the preferred situation the loading was set on 0.60, due to the fact that the remaining number of statements would have been too high when taking a loading of 0.50. For the preferred situation the results were 30 and 29 statements, respectively. The item-total correlation reduced the set of statements to 19 items. Of these reduced sets the Cronbach's alphas ranged from 0.70 to 0.79, while the number of components ranged from six to eight. The explained variance ranged from 56% to 63%. Combining the statements which were included in the questionnaire (after the factor analysis with Varimax rotation and item-total correlation) (Table 1) with the statements that revealed culture gaps, led to a selection of 20 statements with at least two statements per initial Kralewski dimension. Table 2 shows our final CG-Questionnaire.

**Table 1:** Statistical results in chronological order used for the development of the CG-Questionnaire (n = 237)

Order	Physician Present	Manager Present	Physician Preferred	Manager Preferred
Number of questionnaires	166	71	166	71

Number of statements	60	60	60	60

Cronbach's alpha, on 60 statements	0.88	0.87	0.90	0.87

Number of components (factor analysis)	20	20	19	20

Number of statements with loadings above 0.50 in the present situation, 0.60 in the preferred situation	42	43	30	29

Number of statements with a loading above 0.20 after an item-total correlation	26	28	23	19

Cronbach's alpha on the reduced set	0.75	0.70	0.79	0.76

Number of components (factor analysis)	7	6	6	7

Explained variance	59%	61%	56%	63%

**Table 2:** Statements in the CG-Questionnaire, rubricated in accordance with the Kralewski dimensions (* adapted statements from/** added statements to the original Kralewski questionnaire)

Kralewski dimension	1-20	In our hospital...
Collegiality	1	There is a great deal of informal consultation.
	
	7	There is a close collegial relationship among the physicians.
	
	12	There is a strong sense of belonging to the group.

Information emphasis	2	We rely heavily on computer-based information when seeing a patient.
	
	8	We have very good methods to assure that our physicians change their practices to include new technologies and research findings.

Quality emphasis	4	We encourage internal reporting of patient care adverse events.
	
	9	There is an open discussion about clinical failures.
	
	13	We emphasize patient satisfaction.

Management style	5	The business office and administration are considered to be a very important part of our hospital.
	
	16	We expect our administrators to obtain and provide us with information that helps us improve the cost-effectiveness of our patient care.

Cohesiveness	10	There is widespread agreement about most moral/ethical issues.
	
	14	A rapid change occurs in clinical practice among our physicians when studies indicate that we can improve quality/reduce costs.

Business emphasis	15*	When purchasing medical equipment, financial considerations are an important factor.
	
	17**	We only hire an extra physician if he/she is cost-effective.

Organizational trust	3	Our compensation plan rewards physicians who work hard for our hospital.
	
	18**	There is a high degree of trust in the decisions made by the board of directors.

Innovativeness	11	Innovations by our medial doctors are highly publicized.
	
	19**	Our policy plans always mention innovative health care items.

Autonomy	6	There is a feeling that physicians are autonomous but practice in the same organization for support services
	
	20**	The professional autonomy of physicians is an important condition for the quality of health care.

### National data collection with the Culture Gaps Questionnaire, study 3

#### Methodology

Agreement from the general hospitals was needed for the purpose of obtaining a list of physicians and managers to whom the questionnaire could be sent. Therefore we needed the consent and participation of the boards of the hospitals. In our national study, 37 out of a total 86 Dutch general hospitals agreed to participate. These 37 hospitals were spread throughout the country in terms of size and location; therefore we believe we can generalize our findings to the national level. We e-mailed an invitation to all physicians (N = 3941) and managers (N = 680) within the 37 hospitals, who had agreed to participate, to complete the questionnaire on our website. This led to a total N of 4621. After three months the physicians and managers in 11 hospitals who had not responded were reminded with a hard copy sent by post. The remaining physicians and managers in the other 26 hospitals were reminded by e-mail.

The reliability of the total CG-Questionnaire was determined with the Cronbach's alpha. Paired sample T-tests and ANOVA were used to determine significant differences between answers to the statements between physicians and managers. We analyzed differences between physicians and managers, using individual data, pooled over the whole data for the present and the preferred situation and we compared the present and preferred situation. A p-value below 0.05 was considered to be statistically significant. The magnitude and direction of culture gaps were determined with descriptive statistics (mean, standard deviation and the lower and upper bound of the 95% confidence interval).

## Results

In this study we developed a concise, valid questionnaire based on Kralewski et. al. [26,27]. The Cronbach's alphas on all four categories (Table 1) are above 0.70 (meaning a high consistency). With an ANOVA we show that the CG-Questionnaire is able to reveal culture gaps between physicians and managers in Dutch hospitals. The CG-Questionnaire gives a quantitative foundation to the size and content of culture gaps. We pooled the data, but the individual data were used for factor analysis. As a result, the chance of failing to reject statements based on an inappropriate factor analysis, due to pooling the hospitals, is minor.

The average response rate among physicians was 24% (n = 929) and among managers 46% (n = 310), leading to a total response rate of 27% (n = 1239). This response resembled our qualitative and quantitative pilot study (which had a response rate of 35%). As mentioned before, the responding hospitals reflected the national division of urbanization, size and type of hospital. Moreover, our response rates do not deviate much from response rates in comparable Dutch studies [34]. The ratio between physicians and managers from the separate hospitals in the study was comparable to the division of these professions in most general hospitals in the Netherlands. Based on the arguments mentioned above, our results can be seen as reflecting the actual culture gaps between physicians and managers in Dutch general hospitals.

### Culture gaps between physicians and managers

Table 3 presents the results of the main study on culture gaps between physicians and managers on a total group level both for the present and the preferred situation. The Cronbach's alphas of the national data collection was 0.70 and 0.75 for the 20 statements concerning the present situation and 0.76 and 0.79 for the preferred situation.

**Table 3:** Results of the CG-Questionnaire; statement 1 - 20 ANOVA (p-value, significance 0.05), mean and standard deviations of physicians and managers (n = 1239)

	Present			Preferred		
	**Physicians mean** **(SD)**	**Managers mean** **(SD)**	**ANOVA** **p-level**	**Physicians mean** **(SD)**	**Managers mean** **(SD)**	**ANOVA** **p-level**

1	**3.63** (0.904)	**3.70** (0.843)	**0.243**	**3.70** (0.952)	**3.30** (0.875)	**< 0.0001**

2	**2.98** (1.033)	**2.97** (0.995)	**0.924**	**3.75** (0.950)	**4.15** (0.778)	**< 0.0001**

3	**2.13** (0.982)	**2.56** (0.956)	**0.000**	**4.03** (0.901)	**3.61** (0.913)	**< 0.0001**

4	**3.60** (0.960)	**3.74** (0.924)	**0.022**	**4.46** (0.616)	**4.68** (0.526)	**< 0.0001**

5	**3.59** (0.921)	**3.00** (0.908)	**0.000**	**3.29** (0.970)	**3.82** (0.731)	**< 0.0001**

6	**2.55** (0.985)	**3.18** (1.035)	**0.000**	**2.49** (1.174)	**2.01** (0.921)	**< 0.0001**

7	**3.27** (0.969)	**2.77** (0.900)	**0.000**	**4.32** (0.667)	**4.04** (0.665)	**< 0.0001**

8	**2.79** (0.950)	**2.63** (0.825)	**0.007**	**4.20** (0.720)	**4.24** (0.615)	**0.336**

9	**3.12** (0.995)	**2.60** (0.885)	**0.000**	**4.40** (0.685)	**4.45** (0.630)	**0.256**

10	**3.59** (0.830)	**3.40** (0.817)	**0.000**	**4.17** (0.694)	**4.21** (0.645)	**0.403**

11	**3.24** (1.021)	**3.14** (1.016)	**0.126**	**3.75** (0.903)	**4.27** (0.658)	**< 0.0001**

12	**3.11** (0.970)	**3.20** (0.936)	**0.185**	**3.99** (0.730)	**3.99** (0.710)	**0.982**

13	**4.00** (0.816)	**3.79** (0.835)	**0.000**	**4.57** (0.599)	**4.75** (0.481)	**< 0.0001**

14	**3.11** (0.959)	**2.89** (0.880)	**0.000**	**4.28** (0.677)	**4.41** (0.610)	**0.004**

15	**4.36** (0.774)	**4.09** (0.840)	**0.000**	**3.10** (1.027)	**3.72** (0.846)	**< 0.0001**

16	**2.71** (1.076)	**3.20** (1.027)	**0.000**	**4.19** (0.770)	**4.57** (0.586)	**< 0.0001**

17	**3.48** (1.100)	**3.23** (1.050)	**0.000**	**3.26** (1.068)	**3.85** (0.877)	**< 0.0001**

18	**2.94** (0.967)	**3.27** (0.899)	**0.000**	**4.25** (0.739)	**4.39** (0.602)	**0.002**

19	**3.84** (0.829)	**3.90** (0.872)	**0.282**	**4.03** (0.775)	**4.32** (0.671)	**< 0.0001**

20	**3.66** (0.951)	**3.51** (0.827)	**0.013**	**4.24** (0.825)	**3.45** (0.894)	**< 0.0001**

In the present situation 15 out of 20 statements scored statistically significant differences between physicians and managers (ANOVA, p-value < 0.05). Six of these 15 statements were answered in the opposite direction (agreeing (score >3) vs disagreeing (score <3)). As to statement 6 (autonomy of physicians), statement 16 (about provision of information to improve cost-effectiveness) and statement 18 (about trust in decisions made by the board of directors), physicians do not agree, whereas managers do agree. Statement 7 (about collegiality among physicians), statement 9 (about open discussion of clinical failures) and statement 14 (about methods to include new technologies) show the opposite: physicians agree and managers disagree. In the preferred situation there are 16 out of 20 statements scoring statistically significant differences between physicians and managers (ANOVA, p-value < 0.05). The only statement that does not score a statistically significant difference between physicians and managers in both the present and preferred situation is statement 12 (about belonging to the group).

### Culture gaps between present and preferred practices on total group level

As shown in Tables 3, physicians scored differently in 18 out of the 20 statements between the present and the preferred situation. Eight statements even scored differences of over one point. The largest gaps were found in the statements about the compensation plan, the methods that assure practice change to include new technologies, provision of information, and trust in the board of directors. The only two statements not scoring differences were about informal consultation (no. 1) and about the feeling that physicians are autonomous but practice in the same organization for the support services (no. 6). Managers scored differently in 19 out of the 20 statements between the present and the preferred situation. Half of the statements evaluated by the managers scored differences of one point. Examples of the biggest differences are about relying on computer based information, the close collegial relationship among physicians, and the open discussion on clinical failures. The managers scored no differences on the statement about the condition of professional autonomy for the quality of health care (no. 20).

## Discussion

The many large gap sizes in the different culture aspects, uncovered by this study confirm that focusing on rational organizational elements (process analysis, hierarchical and financial structures) may not be sufficient to improve hospital quality. Moreover, the cultural dimension, when thinking about organizational improvement, can be addressed more specifically. Our results support the literature: a focus on cooperation with an explicit eye for intergroup differences should be added to organizational improvement methods [28,30]. With data from the CG-Questionnaire it is now possible to measure the size and content of gaps between physicians and managers in the hospital setting. The CG-Questionnaire enables us to reflect on these differences and provides practical tools for the hospital organization and for future research.

The results of the qualitative pretest and the subsequent quantitative studies confirm the findings in the literature [7,8,11], that there are large differences between physicians and managers. We measured the differences in both how physicians and managers perceive the organizational work practices (present situation) and in the way both groups wish reality to be changed (preferred situation). Differences between both groups in the present and preferred situation can be classified into three categories: (1) culture gaps in the present situation and not in the preferred, (2) culture gaps in the preferred situation and not in the present, and (3) culture gaps in both situations. From an intergroup theoretical point of view these categories require different methodological approaches to improve cooperation between culturally different groups [23]. Clearly, the Intergroup literature can provide us with new insights and methods to enhance cooperation in the professional culture difference setting between physicians and managers. Ben-ari [23] describes three types of methods that can be used to lessen intergroup conflict: the information method, the contact method, and the meta cognition method. All three methods are based on the idea that an increased understanding of the other group enhances cooperation. The three different intergroup methods were applied to the three categories of results of our study, as discussed below.

In our results, three statements scored culture gaps in the first category; differences in the present situation and not in the preferred situation (Table 3). Apparently there is a difference in the way both groups perceive reality. For instance, physicians and managers both agree on the fact that safety and quality in patient care should be guaranteed, but disagree on the level of implementation in the current situation (physicians are more satisfied). In this case physicians and managers do not show an inherent difference in cultures, but differences in perceived practices (based on the professional cultures that both groups have). In this first category the information method from intergroup literature might be effective to lessen the differences between both groups, because the differences are not based on inherent cultures but on differences in perceptions of daily practices. The information method is based on the theory that knowledge about the other group lessens the stereotypes and therefore enhances cooperation. The method consists of providing objective information about the other group [35]. The limitation of the information method is that if the intergroup differences are too large, this approach will not be sufficient on its own [23].

In the second category, four statements scored culture gaps between physicians and managers in the preferred situation and not in the present situation. For instance, publicizing about and mentioning the innovativeness of plans and relying on computer-based registration is preferred more by managers than by physicians. Physicians, on the other hand, prefer more informal consultations (Table 3). This means that both groups adjust their daily practices to hospital reality. If physicians and managers were given the choice to change the daily practices individually, physicians would change it in a different way than managers would. An intervention that might help in this case could be a method based on Allport's contact theory [22]. This theory has been applied in many studies and has proven its value [20]. The contact theory maintains that contact between members of different groups enhances cooperation. There are four conditions to be met for the contact to be effective: having common goals, no competition between groups, support by the authorities, and equal status [22]. For example, the intervention could be a project in which both groups are given an assignment to mutually design a plan that integrates preferred situations for both groups.

The third category shows 12 statements that score culture gaps in the present and the preferred situation; these statements reveal the most imminent aspects of the culture gaps. Therefore, these are presumably the most difficult aspects to change within a hospital organization. Physicians' values of the professional autonomy and collegiality among physicians are higher than among managers. Consequently, the physicians scored even higher in the preferred situation. Managers value the registration of adverse events and the trust in the decisions made by the Board of Directors was higher in the current situation compared to the physicians. Managers want it to be even higher in the preferred situation. The situation in which both the preferred and the present situation are different, can be seen as a persisting intergroup conflict situation. From intergroup literature, the third approach, meta cognition, facilitates the overcoming of cognitive obstructions, preventing openness towards information about the other group. A training program could provide insight into the way cognition works and therefore the awareness of ones own prejudices towards the other group. This, in turn, provides the opportunity to reflect on the sources of prejudice. Insight into meta cognitive processes can change people's perceptions and behavior [36].

Methodologically, in the process of transforming Kralewski's questionnaire into the CG-Questionnaire, the extra dimension added to the initial nine dimensions was not confirmed to be a one-dimensional construct and, therefore did not add information. Therefore we decided not to include these additional six statements in that dimension into further data analysis, nor in the CG-Questionnaire. Statistically, we could not confirm Kralewski's nine dimensions. We checked whether the scores in the factor analysis with Varimax rotation were caused by the additional statements, when compared to the original Kralewski questionnaire [26], but that was not the case. We repeated the factor analysis with Varimax rotation on the results of the original, but translated statements. This also did not show the initial nine dimensions. The following factors could have caused this. First, our goal was different from the initial goal of Kralewski's questionnaire; the CG-Questionnaire intends to measure the size and content of culture gaps between physicians and managers. Kralewski's questionnaire is used to differentiate between medical groups. Second, the Dutch hospital system differs from the situation in the USA with exclusively non-profit type organizations and more pronounced central regulation. The socio-economic structure possibly influences the way physicians and managers (co)operate. This is also why we altered two statements earlier and added one statement (to the dimension business emphasis) before the pretest; these three statements have been incorporated into the final set of 20 statements comprising our CG-Questionnaire. Although statistically we could not confirm the dimensions of Kralewski et. al., the CG-Questionnaire meets our research objective because data gained with the questionnaire gives us leads as practical tools to enhance cooperation between both groups. The high Cronbachs alphas (0.70 and 0.75 present, 0.76 and 0.79 preferred) of the CG-Questionnaire show that the statements in the questionnaire form a coherent construct.

Despite the differences between the goal of our study and Kralewski's goal, and the different national settings of the researches, the underlying issues in health care (such as the need for more efficacy, safety, and quality of care) are also relevant in health care elsewhere [3,7,8,28]. Therefore, it could be of interest for hospital organizations outside of the Netherlands to apply the CG-Questionnaire. The applicability and validity should be studied before using the CG-Questionnaire in other countries. However, there are a number of countries in which the validation process would be easier because of the similarity in the way in which their health care is organized. These countries are for example: Germany, Belgium, France and the Scandinavian countries.

Further research could focus on the relation between different gap sizes between physicians and managers and the effectiveness of the cooperation; it is possible that a small gap could be hypothesized as causing possible productive friction. Either studies covering large numbers in cross sectional designs, or longitudinal studies using interventions, could lead to new and creative insights to significantly enhance the effectiveness of the cooperation.

## Conclusions

The results of our study confirm the existence of the presumed latent conflict between physicians and managers and show that, below the surface of the daily practices, the relationship between (members of) both groups is tense, leading to suboptimal cooperation. This might decrease hospital performance, and could ultimately harm patients. When implementing change, hospitals could use the CG-Questionnaire to gain data on the content and size of culture gaps between physicians and managers in order to better substantiate the chosen methods used for organizational improvement. Combining results from the present and preferred culture gaps, we arrived at three categories of increasing complexity. We linked these categories to the three methods described in the intergroup literature to help develop the interventions. Interventions based on intergroup literature (enhanced information, contact and ultimately meta cognition) could help improve the cooperation between physicians and managers. At the very least, our findings will increase the awareness of the importance of tension, which is kept below the surface, and ultimately hindering effective cooperation. Our results may stimulate research into the relation between the size and content of culture gaps that hinder cooperation and affect the performance of hospitals.

The adjusted questionnaire and the full rotated matrix of the factor analysis can be obtained from the authors by email.

## Competing interests

The authors declare that they have no competing interests.

## Authors' contributions

HAHJK contributed to the design, data retrieval, analysis and writing of the paper. SS contributed to the design, analysis and writing of the paper. NM contributed to the analysis and writing of the paper. CPMW contributed to the design and writing of the paper. WHvH contributed to the design, analysis and writing of the paper. All authors read and approved the final manuscript.

## Pre-publication history

The pre-publication history for this paper can be accessed here:

http://www.biomedcentral.com/1472-6963/10/86/prepub
